# Multi-criteria decision analysis for health technology assessment: addressing methodological challenges to improve the state of the art

**DOI:** 10.1007/s10198-019-01052-3

**Published:** 2019-04-20

**Authors:** Mónica D. Oliveira, Inês Mataloto, Panos Kanavos

**Affiliations:** 1 CEG-IST, Universidade de Lisboa, Avenida Rovisco Pais, 1049-001 Lisbon, Portugal; 2 Department of Health Policy and Medical Technology Research Group, LSE Health London School of Economics and Political Science, Houghton Street, London, WC2A 2AE UK

**Keywords:** Multi-criteria decision analysis, Health technology assessment, Systematic review, Methodological quality, Methodological challenges, MCDA modelling, I (Health education and welfare), I10 (General), I18 (Government policy regulation public health), C44 (Operations research statistical decision theory), D80 (General)

## Abstract

**Background:**

Multi-criteria decision analysis (MCDA) concepts, models and tools have been used increasingly in health technology assessment (HTA), with several studies pointing out practical and theoretical issues related to its use. This study provides a critical review of published studies on MCDA in the context of HTA by assessing their methodological quality and summarising methodological challenges.

**Methods:**

A systematic review was conducted to identify studies discussing, developing or reviewing the use of MCDA in HTA using aggregation approaches. Studies were classified according to publication time and type, country of study, technology type and study type. The PROACTIVE-S approach was constructed and used to analyse methodological quality. Challenges and limitations reported in eligible studies were collected and summarised; this was followed by a critical discussion on research requirements to address the identified challenges.

**Results:**

129 journal articles were eligible for review, 56% of which were published in 2015–2017; 42% focused on pharmaceuticals; 36, 26 and 18% reported model applications, issues regarding MCDA implementation analyses, and proposing frameworks, respectively. Poor compliance with good methodological practice (< 25% complying studies) was found regarding behavioural analyses, discussion of model assumptions and uncertainties, modelling of value functions, and dealing with judgment inconsistencies. The five most reported challenges related to evidence and data synthesis; value system differences and participant selection issues; participant difficulties; methodological complexity and resource balance; and criteria and attributes modelling. A critical discussion on ways to address these challenges ensues.

**Discussion:**

Results highlight the need for advancement in robust methodologies, procedures and tools to improve methodological quality of MCDA in HTA studies. Research pathways include developing new model features, good practice guidelines, technologies to enable participation and behavioural research.

## Background

In a context of increased ageing and epidemiological change, technological advances, increasing patient expectations and budget constraints, health systems are facing considerable challenges to improve access to innovation, enhance rationality in decision-making processes, and improve their efficiency and effectiveness. In this context, health technology assessment (HTA) is playing a critical role by bringing together evidence to help healthcare decision-makers in understanding the relative value of health technologies [1, 2].

As a multidisciplinary field involving theoretical and practice-oriented research to assess the direct and indirect consequences of health technology use [3], HTA is currently challenged in various ways. First, despite the increased use of HTA in many jurisdictions [4], a number of new health technologies—for instance biomedical technologies—are increasingly approved and adopted based on limited evidence on safety and effectiveness, with assessment under real-world conditions being rare, and technologies being used for little or no additional health gain [5]. Second, effective use of HTA requires the involvement of health stakeholders and the implementation of HTA findings, which is far from happening on a routine basis [6]. Third, HTA needs to resolve issues related to the deployment of existing evaluation methods and processes (with some methodological issues, such as the extent to which cost-effectiveness analysis is appropriate to evaluate all types of health technologies, remaining unresolved) [6, 7] and to address the lack of good quality evidence for many evaluation contexts and technologies [8]. Fourth, for HTA to have an impact there is a need to link and align decision processes at distinct health system levels, as decisions at these levels inter-relate [8]. Nevertheless, health technology decision-making by HTA agencies, hospitals and other organisations often remains unconnected. Finally, HTA needs to go beyond the evaluation of pharmaceuticals, with literature acknowledging that other technologies (such as medical devices and health information systems), or, indeed, the broader space of health care interventions, place additional challenges from a methodological and practical perspective [9].

At the core of HTA is the task of measuring additional value, aligned with the spirit that ‘you manage what you value’ [10] and the promotion of value for money in health systems [2]. Most literature in the health field has focused on traditional techniques based on the measurement of value as captured by comparative effectiveness, with effectiveness being centred on health outcomes or on health utilities. Emerging literature, however, has been exploring alternative and more comprehensive ways to measure value. In line with views that other dimensions are relevant for decision-making regarding health technology adoption (for instance equity and innovation), with a sense of inevitability in considering other criteria than clinical-and-cost-effectiveness [11], and with evidence suggesting that HTA agencies consider in practice other aspects in adoption, reimbursement and pricing decisions [12, 13], several studies have been exploring the use of multi-criteria decision analysis (MCDA) concepts in HTA.

Framed within decision analysis, MCDA operates within a paradigm of rationality, as defined by Simon [14], offering “a philosophy, articulated by a set of logical axioms and a methodology and collection of systematic procedures, based on those axioms” [15]. As a sound approach with theoretical foundations, MCDA can be seen as “an extension of decision theory that covers any decision with multiple objectives, a methodology for appraising alternatives on individual, often conflicting criteria, and combining them into one overall appraisal” [16]. As a field it operates as a toolbox offering a wide range of concepts, models and tools and a clear framework for thinking about resource allocation decisions and a common language [17].

The potential of MCDA in the health field has been discussed widely; such discussion has led to two taskforce reports from the International Society for Pharmacoeconomics and Health Outcomes (ISPOR) [18, 19] and to several literature reviews [20–23]. The usefulness of MCDA in HTA has been supported in a number of other studies [11, 24]. Clear arguments provided for its use have been its alignment with value-based health care [25]; its encompassing nature and ability to account for health stakeholder preferences and values [26]; its transparent and synthesis reporting format [27]; its contribution in helping decision-makers to understand technology value and data gaps [21] and differences between evidence and value judgments [19]; its easily understandable outputs [24]; and the underlying link with the accountability for reasonableness principle [28]. MCDA has been recalled as a commonly used approach for priority-setting [29], and a number of organisations, including the European Medicines Agency and the Institute for Quality and Efficiency in Health Care (IQWiG) in Germany, have shown interest and explored the use of MCDA methods in drug regulatory science and HTA, respectively [30].

Although MCDA provides theoretically sound methods to balance costs, benefits and risks, and multicriteria models have been seen as intuitive by evaluators, several studies [19, 30–33] have pointed to a number of shortcomings: first, publications under the ‘MCDA in HTA’ umbrella have sometimes used methods without a sound basis or made an inadequate use of existing methods [32, 33]. Second, studies have recognised the need to develop methods, knowledge and guidelines in the area, for instance, to address the use of inappropriate procedures for weighting (not accounting for attribute ranges [33] leads to the most common mistake reported in decision analysis literature [34]), a lack of testing for the adequacy of using additive model structures [32], and the need for developing methodological and practical guidelines to assist MCDA users [19]. Third, most articles in the literature have reported pilot and exploratory studies of MCDA in HTA, with few studies reporting successful implementations of MCDA models and results in the context of HTA, and with some studies reporting cognitive difficulties from participants in the process of model-building [19].

Despite these shortcomings, there is no comprehensive analysis of the extent to which methodological issues related to the application of MCDA in the context of HTA affect the credibility and policy-usefulness of published literature, and the range of challenges and limitations that need to be addressed by MCDA in this context. In light of this, the aim of the study is fourfold: first, to provide a critical review of published studies in the field; second, by applying a framework, to analyse the quality of MCDA studies in the context of HTA from a methodological perspective, as distinct from a policy-perspective that could have been adopted as an alternative; third, to summarise challenges and limitations reported in relevant studies; and, fourth, to reflect on how MCDA applied in the context of HTA can overcome these challenges and limitations. The study contributes to the literature in four ways: first, it provides a critical appraisal of studies applying MCDA in the context of HTA, their scope and trends; second, it defines and applies an approach to assess the methodological quality of MCDA model applications; third, it informs on which modelling steps improvements are needed; fourth it identifies and reports on a number of methodological challenges and limitations that need to be addressed and discusses how future studies can overcome these challenges.

The study is organized as follows: the “Methods” section outlines the review protocol and the methods used in the analysis of eligible studies, discusses the methodological quality framework and the process followed to collect and summarise challenges and limitations. The next two sections report the results, and discuss the results and reflect upon how MCDA in HTA can address the identified challenges and limitations. Finally, the last section concludes.

## Methods

### Review protocol and studies’ analyses

We conducted a systematic search on 18 September 2017 on the databases: PubMed, EBSCO, Web of Science, ScienceDirect and SAGE. A search protocol was developed and applied on the title and abstract fields, with a keyword combination recognising the range of terminological variations regarding MCDA and HTA (for instance related with similar designations such as multi- vs. multiple, criteria vs. attribute, decision analysis vs. decision aiding vs. decision theory, HTA vs. benefit-risk assessment). The search protocol, including all combinations used is shown in Appendix A. The literature search was restricted to journal articles and book chapters written in English, with no time constraints being applied. Preferred Reporting Items for Systematic Reviews and Meta-Analyses (PRISMA) [35] guidelines were taken into consideration in the development of the study.

Duplicates were removed from the collected studies, and titles and abstracts were screened by two reviewers (MO and IM) by applying the following predefined inclusion criteria: studies would have to discuss, develop or review the use of multi-criteria analysis (focusing on aggregation approaches only, notably following the strategy of first modelling components of value explicitly and then aggregating these components) for the evaluation of health technologies administered by the health sector. The review took a broad perspective on MCDA as a strict view would require considering only MCDA studies respecting decision theory principles. Studies explicitly structuring the criteria were included if they indicated to be a step towards MCDA model development. Similarly, studies using non-aggregation approaches (e.g. discrete choice experiments) were included only if they provided data to be used as an input to multicriteria aggregation models. Finally, other systematic literature reviews identified as part of the search strategy were included in the eligible studies.

The following exclusion criteria were applied: studies focusing on technologies not strictly classified as health technologies or not administered by the health sector, such as water cleaning technologies, medical waste, solid waste, environmental health, pure or general risk assessment; studies in which multiple criteria were not directly applied to a HTA context, including safety and efficacy assessment used in studies other than marketing authorisation; retrospective evaluation studies (strictly inferring criteria from previous decisions); decision quality and behavioural decision analysis studies; clinical reasoning and descriptive clinical decision-making studies; studies that presented a minor MCDA component, namely those having MCDA combined with other modelling approaches and those discussing several evaluation approaches, but with minor MCDA explanation or discussion, or with little more than mentioning MCDA as a technique among other evaluation techniques; studies recommending the use of MCDA without a detailed discussion about its rationale; MCDA patient preference assessment studies if they were not designed to directly compare health technologies (e.g. those to build quality of life utility scores); studies in languages other than English; and studies corresponding to conference proceedings were excluded if not adhering to the implemented protocol.

The full-text of the articles considered eligible was obtained from public sources or requested from the authors if not available otherwise. Articles for which the full-text was not made available, were removed. Supplementary articles and book chapters that were not found through the protocol but were identified along the initial review of studies and deemed to be within the protocol scope and published before the end of 2017 were added.

### Analysis of eligible studies

The studies included for systematic review were classified with regards to the time of publication, type of journal, country of study, health technology focus and type of study. Since the number of studies covering the scope of this review has been increasing considerably since 2008 and one aimed at capturing recent trends (while avoiding periods with small numbers of studies, year fluctuations and uncomparable periods), periods of 3 years from 2009 onwards were considered, resulting in four time windows: up to 2008, 2009–2011, 2012–2014, 2015–2017.

Regarding the health technology focus, the following categories were considered: pharmaceuticals, vaccines, medical devices, nanotechnologies, general health technologies (e.g. medical devices or medical equipment), and health interventions (e.g. assessing tobacco control vs. promoting physical activity in the workplace vs. prescribing to control blood pressure). Studies not focusing on a specific type of health technology were classified as general health technologies; and studies centred on health interventions, strategies or programmes to promote individual or community health benefits, health equity or healthy behaviours were classified as health interventions.

Publications were also classified according to their methodological/conceptual/theoretical or practical/empirical focus: clinical, non-clinical (but health related), Operational Research/Management Science or interdisciplinary.

Regarding the country of study, studies were classified according to the institutional location of the first author.

Regarding the type of study, studies were categorized according to their main focus in the following categories: methodological or conceptual frameworks, analysis of issues, systematic literature reviews, structuring criteria studies, modelling approach studies, or model applications. Frameworks were defined as studies suggesting the use of MCDA methods and tools for HTA and defining guidelines or procedures for its use; analysis of issues were studies calling attention and discussing issues related to the development and use of MCDA in HTA; structuring criteria studies were those analysing the evaluation dimensions to be used within the scope of MCDA modelling; modelling approach studies were those developing MCDA approaches to address HTA issues; finally, model applications were studies reporting MCDA evaluation models to compare health technologies in practice. Within each type of study, the focus of the reported research was analysed.

### Framework for analysing methodological quality

As no established approach for assessing the methodological quality of MCDA studies has been reported [36], this study developed the PROACTIVE-S approach for this purpose, as an enhancement to the PROACTIVE (Problem, Reframe, Objective, Alternatives, Consequences and Chances, Trade-Offs, Integrate, Value, and Explore and Evaluate) approach [37] that in itself was inspired in the PrOACT-URL approach (PROblem, Objectives, Alternatives, Consequences, Trade-offs, Uncertainty, Risk attitudes, and Linked decisions) [38]. PROACTIVE specifies each of these components as a modelling step in which some tools may be used, while it builds upon the eight elements for sound decision processes defined in the Smart Choices PrOACT-URL approach [38]. Both PROACTIVE and PrOACT-URL are explicit processes that require a clear and deep understanding by decision-makers before they commit to a decision [38] and are aligned with a value-focused thinking perspective, which is specifically useful to: (a) guide strategic thinking, (b) facilitate collective decision-making, (c) uncover hidden objectives, (d) direct the collection of information and (e) improve communication [34]. In comparison to PrOACT-URL, PROACTIVE makes more explicit the role of evidence, values, uncertainty and integration of these components [37], which are deemed particularly relevant in the context of HTA.

To produce an approach that can be used for the assessment of methodological quality of MCDA studies according to good practice considerations, PROACTIVE-S was developed (see Table 1) by adapting, adjusting, enhancing and improving PROACTIVE by:

**Table 1:** Defining the PROACTIVE-S approach to analyse the methodological quality of evaluation models reported in the “MCDA in HTA” literature. Source: the authors

PROACTIVE-S step	Step scope	Sub-step good practice considerations—the extent to which the study…	Sub-step abbrevia-tion
Problem	Define the problem	…describes the evaluation context, the decision goal and reflects upon the type of evaluation problem (decision problematique) [40]	P1
Reframe	Reframe relevant multiple perspectives	…considers/discusses the perspectives of relevant stakeholders and key players, clarifies the perspective of the problem owner [203] and discusses whose views should be considered in model building [43]	R1
Objective	Focus on the objectives	…focuses on the objectives to be achieved [34] (rather than on focusing upon indicators and criteria)	O1
Alternatives	Consider all relevant alternatives	…defines and discusses the relevant health technologies to be evaluated and linked decisions [38]	A1
Consequences and chances	Model consequences, uncertainty and lack data	…assesses relevant consequences in adequate attributes that comply with required properties (measurable, operational, understandable) [34, 47] and organises options consequences into a performance matrix [18]	C1
		…discusses data sources and issues [42], as well as consequences’ uncertainty [41]	C2
Trade-offs	Understand value trade-offs	…discusses trade-offs among competing objectives or criteria [34]	T1
Integrate	Integrate the evidence and values	…distinguishes between evidence, options’ performance and value information in model building [18]	I1
Value	Build a value model and maximize value	…discusses model respect for exhaustiveness, non-redundancy and non-overlap properties in additive models or other relevant properties for other models [34]	V1
		…discusses preference independence conditions and presents the underlying model structure (p.e. additive model formula) [39, 41]	V2
		…uses methods for model building that comply with multiattribute decision theory [16, 39]	V3
		…defines mechanisms to detect and correct inconsistencies [19, 48]	V4
		…uses procedures to model value functions [34, 41]	V5
		…uses weighting procedures that utilize weighting references [34], explaining the rationale for choosing those references [40, 48]	V6
Explore and Evaluate	Explore assumptions and evaluate uncertainty	…explicits model assumptions and the relevant uncertainties for the evaluation context (p.e. imprecise consequences, variable consequences, quality of evidence, structural uncertainty and judgmental uncertainty) [19]	E1
		…tests the consequences of model assumptions and uncertainty (e.g. sensitivity, robustness and/or scenario analyses) [45]	E2
		…discusses model validation and requisiteness [45, 49] and questions model results [41]	E3
		…uses computer technology to display results and motivate discussion [50, 51]	E4
Social	Build and implement a socio-technical design	…the study is replicable by making explicit: model building participants, participatory scope and format, participatory timeline, and protocols of questioning [48, 52]	S1
		…takes into consideration behavioural aspects, such as cognitive burden and potential biases [53–55]	S2
		…promotes participants reflection and iterativeness in model development while promoting consensus [45] and/or reflects in ways to combine participants’ assessments [52]	S3

- adjusting some steps and specifying each step into a set of sub-steps that detail good practice considerations based on multi-criteria decision theory, value measurement and value focused thinking literature [16, 34, 39–41] and studies reflecting good practice aspects regarding the use of MCDA in health [18, 19, 42–44];
- adding a “social step” (S) to ensure rigor, reliability and potential replicability of MCDA in HTA studies and understand participants’ attitudes and consensus regarding the constructed models. Adding this step is aligned with the view that MCDA modelling inherently follows a socio-technical approach that “combines technical elements of MCDA with social aspects of decision conferencing, resulting in a tested approach to working with key players that creates shared understanding of the issues, a sense of common purpose and commitment to the way forward” [45] and builds upon the socio-technical design principles proposed by Cherns [46]. Accordingly, social processes—that can encompass face-to-face, non-face-to-face processes or a combination of both—need to be properly designed and tested within MCDA for HTA.

The following steps from PROACTIVE are adjusted and divided into several sub-steps, and the “S” extra step is added:

- Consequences and chances need to consider not only aspects related to the proper construction of attributes [34, 47] that serve to characterise technology performance [18], but also to discuss data sources, issues [42] and uncertainty [41].
- Value measurement requires considering that the properties needed for using an additive model properties and model structures are sustained and reflected upon [34, 39, 41], that methods complying with multi-attribute decision theory are adopted [16, 39], that mechanisms to detect and correct inconsistencies are utilized [19, 48], that procedures to model value functions are used [34, 41], and that weighting procedures making use of weighting references are used [34] and the rationale for choosing those references is explained [40, 48].
- Explore and evaluate requires reflecting upon model assumptions and uncertainties [19] and testing the consequences of assumptions and uncertainties [45], discussing model validation and requisiteness issues [45, 49] and questioning model results [41], and using decision support technology (e.g. IT) to display results and motivate discussion [50, 51].
- The added social component (S) requires that the social process is described in detail [48, 52] (for instance for replicability and to enable result interpretation), takes into consideration behavioural aspects [53–55], promotes participants’ reflection and consensus [45] and/or reflects how to combine participant assessments [52].

### Protocol for identifying limitations and challenges

To collect information on limitations and challenges concerning the use of MCDA models in the context of HTA, articles were searched for the words “limitat*”, “challenge*”, “barrier*”, “difficult*”, “pitfall*”, “disadvantage*”, “accept*”, “implement*”, and “concern*”. Reported issues related to the use of MCDA in HTA were collected, for instance, general HTA concerns not specifically related to MCDA were not considered, and subsequently were clustered with similar or related limitations and challenges. The 12 most frequently cited clusters of limitations and challenges were summarised and studies expressing these concerns were identified.

## Results

### Protocol results

**Fig. 1:**
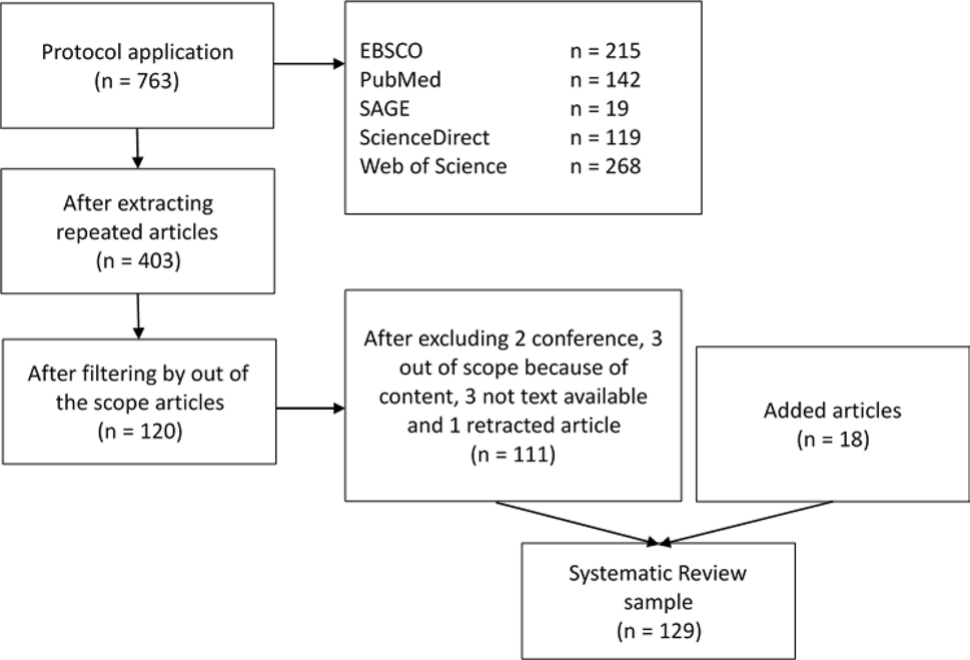
PRISMA flowchart describing study selection

The search protocol yielded a total of 763 studies, of which 403 remained following the removal of duplicates. Screening at title and abstract level resulted in the elimination of 283 studies, leaving 120 studies to assess in full-text level. Among these, three full-texts could not be obtained (from Thai and Polish journals), three studies were considered out of scope because of content, two studies were conference proceedings and one article had been retracted. A further 18 studies that were deemed relevant (identified along the initial review of studies and deemed to be within the protocol scope) were added, resulting in a final sample of 129 studies included in the systematic review. The results from the literature selection process are presented as a PRISMA flowchart in Fig. 1.

### Study characteristics

**Fig. 2:**
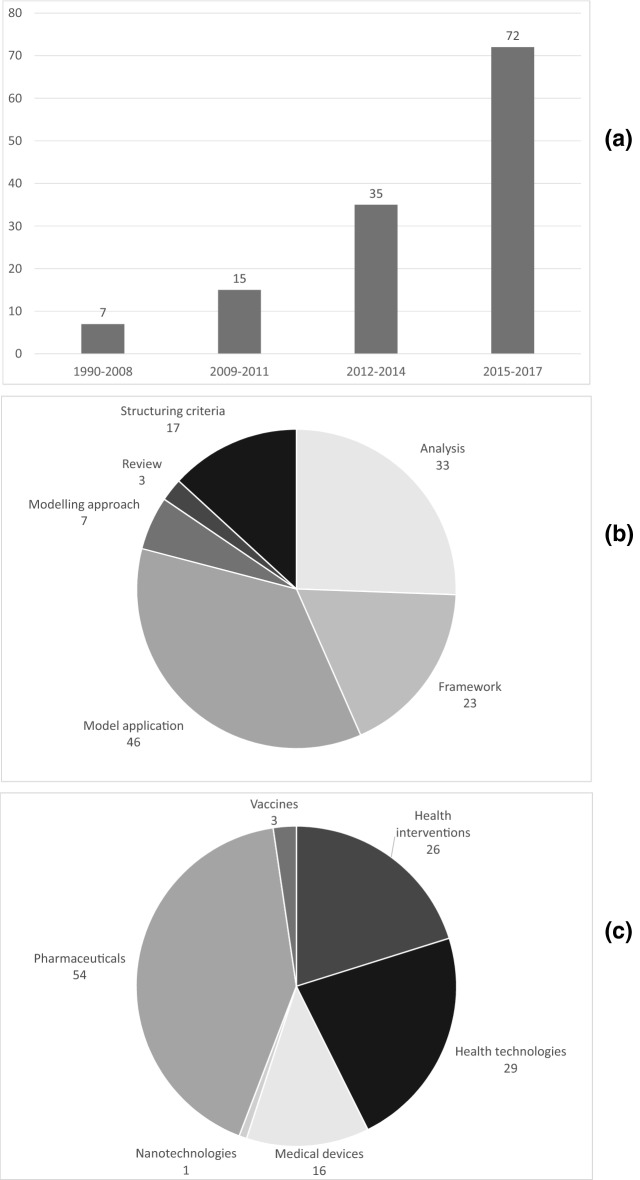
**a** Number of article publications over time; **b** number of publications by type; **c** number of publications according to health technology focus. Source: the authors from the literature

Period of publication: The 129 studies meeting the inclusion criteria were published between 1990 and 2017, with an upward publication trend being observed (Fig. 2a). Only 5% of the studies (seven studies) were published up to 2008, whereas the period between 2015 and 2017 accounted for 56% of the study sample (72). In the interim, 15 and 35 studies were published in the 2009–2011 and 2012–2014 periods, respectively.

Type of publication: The studies were published in 59 different journals that cover a wide range of perspectives. *Value in Health* published the largest number of studies (17), followed by the *International Journal of Technology Assessment in Health Care* (9) and *Pharmacoeconomics* (8). Sixty-eight percent of all studies were published in health (non-clinical), 26% in clinical, 5% in operational research/management science and 1% in interdisciplinary journals.

Study country: First author institutions spanned across 27 countries, with the most frequent first author institutions being located in the UK (30 studies), followed by Netherlands (17), Canada (17), US (16), Germany (8), Italy (7) and Hungary (4). Twenty other countries accounted for three or less studies each.

Health technology focus: Based on Fig. 2b, 44 studies (42%) focused on pharmaceuticals (the majority analysing pharmaceuticals in general, rather than pharmaceuticals for a specific therapeutic indication), with 29 studies investigating general health technologies, 25 studying health interventions, 16 studying medical devices (most of them comparing different devices), 3 researching vaccines (all of them exploring relevant criteria to assess vaccines), with one considering a nanotechnology.

Type of study: According to their main focus, 46 studies (36%) developed models to evaluate health technologies in practice; 33 (26%) analysed MCDA implementation issues; 23 (18%) proposed frameworks to support MCDA in HTA implementation; 7 (5%) explored modelling approaches; and 3 (2%) provided a systematic literature review (Fig. 2c). The content in each of these study groups is discussed below.

#### (a) Model application studies

The 46 model application studies evaluated pharmaceuticals (24 studies), medical devices (12), health interventions (9) and general health technologies (1). Pharmaceuticals constituted the subject matter of investigations in the following disease areas: rare diseases (none of which contained orphan cancer indications) [56–60], cancer [61–63], depression [64, 65], cerebrovascular diseases [66, 67], pain-relief [68, 69], age-related macular degeneration [70], overactive bladder [71], idiopathic short stature [72], Turner syndrome [73], psoriasis [74], hypercholesterolemia [75], chronic obstructive pulmonary disease (COPD) [76] and relapsing–remitting multiple sclerosis (RRMS) [77]. Two studies developed models to compare pharmaceuticals targeting several diseases [27, 78].

Medical device studies included imaging, surgical and screening approaches, notably, CT, MRI and ultrasound devices [79], MRI equipment [80], imaging techniques, software platforms for cerebrovascular diseases [81], photoacoustic mammoscope technique for breast cancer diagnosis [82], surgical approaches for cam femoroacetabular impingement [83], non-fusion surgery approach for adolescent idiopathic scoliosis [84], surgical robotic innovations [85, 86], reusable pedicle screw instrument kit for lumber arthrodesis [87], a pulmonary heart sensor [88], drug eluting beads for transcatheter arterial chemoembolization [89] and a screening test for cervical cancer [90].

Evaluated health interventions included public health programmes [91], primary care programmes [92, 93], community care programmes [94], screening strategies [95], mental health services [96], smoking cessation interventions [97] and types of medical care to be covered [98, 99]. One study evaluated both pharmaceuticals and surgical technologies for priority setting purposes [100].

Most applications in this group (34 studies, 74%) aimed to select the most valuable technology, although other purposes have been reported: ranking technologies (6), allocating available resources to technologies (5), and assigning to reimbursement categories (1). With regards to social processes, 39 studies (85%) reported the use of participative methods, 32 (70%) adopted face-to-face approaches for model-building, including decision conferences and workshops, whilst 7 (15%) used web-based formats; 10 (22%) used questionnaires/surveys, 2 (4%) each used interviews and Delphi processes; 7 (15%) studies developed models based upon authors’ opinion or did not detail if and how participatory processes took place; 18 (39%) studies dealt with aggregation of individual answers within modelling.

The most frequently used procedures for weighting criteria were the analytical hierarchy process (AHP) (12), quantitative swing weighting (9), point-scaling (8), 100-point allocation (4), and qualitative swing weighting with the measuring attractiveness by a categorical-based evaluation technique (MACBETH) (3); other procedures included the simple multi-attribute rating technique (SMART), SMART/SMARTS (SMART with Swings)/SMARTER (SMART Extended to Ranking), Borda points, equal weighting and weighting calibration according to fatal events. Studies reported building value scales with point systems (11 studies), direct rating (7), MACBETH (3), AHP (2), and selecting functions (3), including one selecting linear value functions; 18 studies either did not provide information about value scoring issues or implicitly opted for not modelling value scales. Only one study reported a non-additive model, having used a multiplicative model [96]. Five studies reported that results from MCDA modelling had practical consequences for decision-making.

#### (b) Analysis studies

The 33 studies in this category discussed a range of MCDA issues related to HTA adoption, notably (a) raising methodological issues, (b) analysing the relevance of MCDA in HTA, (c) providing a critique on the use of MCDA in HTA and (d) discussing aspects related to its practical use in the HTA context.

With regards to methodological issues on the use of MCDA in HTA, studies addressed a range of issues: first, they provided an overview of MCDA methods and their potential for supporting transparent and consistent health care decision-making [18]; second, they analysed the most common types of MCDA models applied in HTA and identified practical issues in its use [24]; third, they discussed requirements and defined steps for a Universal Methodology for Benefit-Risk Assessment (UMBRA) [101]; fourth, they compared MCDA with other methods for considering equity–efficiency trade-offs in assessing breast cancer control options [102], for evaluating medical devices and imaging technologies [103] and for comparing patient preferences [104]; fifth, they discussed MCDA as a method to integrate economic evidence into clinical practice guidelines [33]; sixth, they described (structured) Evidence to decision frameworks as an alternative or as complementary to MCDA models [105]; and, finally, reported 16 best practice principles for MCDA practice in HTA, with emphasis to facilitated group modelling [44].

Regarding the relevance of using MCDA in HTA, studies, first, discussed the use of MCDA methods to overcome barriers in priority setting, particularly by accounting for the views of healthcare stakeholders [106]; second, they recommended MCDA for dealing with stroke interventions requirements [107]; third, they discussed MCDA usefulness in the context of personalized healthcare by dealing with nuanced and context-specific information that decision-makers would typically require [108]; fourth, they suggested MCDA as a comprehensive tool for dealing with distinct criteria in priority setting or for designing healthcare coverage packages [109]; fifth, they suggested MCDA to value pharmaceuticals [110], arguing for its specific suitability in the rare diseases context [111, 112]; sixth, they discussed the potential role of MCDA to implement a hedonic-pricing approach by bringing together multiple perspectives on the benefits (and potential harms) of medical technologies [113]; seventh, they suggested MCDA to operationalise value-based pricing and aggregate elements of value that are not well represented by weighted quality adjusted life years in a pragmatic way [25]; eighth, they discussed the relevance of including evidence on patient preferences in MCDA [114]; ninth, they suggested MCDA as a tool to include all the relevant criteria that impact on decision-making within transparent processes in Canada [115] and analysed the benefits and challenges regarding its use in that context [116]; tenth, they suggested MCDA to explicitly model non-economic criteria in pricing and reimbursement decisions in Central and Eastern Europe [117]; and, finally, they suggested MCDA as a methodological approach to increase efficiency, rationality and legitimacy in resource allocation decisions [118].

A sizable group of studies provided a critical appraisal on the use of MCDA in HTA. Studies in this group, first, argued that MCDA can make decision-making too complex or too mechanistic, removing the element of deliberation [119]; second, they showed that MCDA, similarly to other economic evaluation methods, failed to incorporate opportunity costs [120]; third, they alerted for methodological flaws in current applications [121]; fourth, they alerted on the risk of MCDA adding complication since its influence on decision-makers and stakeholders was described as not clear in pharmaceutical pricing and reimbursement contexts [122]; fifth, they suggested the treacle test (can a winning intervention be incompletely ineffective?) and the smallpox test (can a winning intervention be for a disease that no one suffers from?) to raise questions about the adequacy of evaluation model structures reported in the field [32]; and, sixth, they raised issues about the validity and reliability of MCDA for evaluating pharmaceuticals and providing suggestions for improving methodological quality [31].

Finally, with regards to the practical use of MCDA in HTA, studies concluded positively upon first experiences of applying MCDA to prioritize health interventions [123]; discussed the implementation of HTA in Latin American countries, concluding that although MCDA has been applied in few cases, most health stakeholders declared preferring its use [124]; discussed its limited use in Hungary, but the relevance for its development [125]; and discussed that stakeholders in a case study favoured structured approaches to integrate patient views [126].

#### (c) Framework studies

Twenty-three studies explored the use of MCDA methods and tools for HTA and defined related guidelines or procedures in multiple decision-making and evaluation contexts. A first group of framework studies focused on the use of MCDA in HTA for specific health technologies: to assess new medical technologies [26, 127]; to evaluate pharmaceuticals while focusing on new pharmaceuticals [30, 128], social values [129], (older) well-established pharmaceuticals whose benefit–risk balance may have changed [130] and drug or dose comparisons [131].

A second group of framework studies was developed for specific purposes, exploring one of the following areas: to apply MCDA in clinical decision-making when clinical consensus regarding clinical criteria and weights is required [132]; to inform radiology guideline development [133] and disease management programs [134]; to select an optimal nanomaterial for a given medical application [135]; to inform drug ranking for formulary listing in low-income countries [136]; to propose MCDA in HTA for middle-income countries [137] and to critically reflect upon that proposal [138]; to inform a wide range of decisions, e.g. approval, guidelines and pricing/reimbursement [43]; to evaluate statins for primary prevention of cardiovascular disease [42]; and to select criteria to be used in HTA [139, 140].

A third group of framework studies focused on principles, methods and processes, aiming to (a) integrate MCDA and accountability for reasonableness principles to support HTA agencies and promote legitimacy in reimbursement recommendations [28]; (b) account for good practice considerations to implement MCDA in healthcare (not specific to HTA) [19]; (c) support deliberative processes behind decision-making by dealing with data analysis, synthesis and validation by experts in general [141], and for rare diseases [142] in particular; and (d) prioritize health technologies based on value for money concepts and under a limited budget [143].

#### (d) Structuring criteria studies

Studies structuring criteria aimed at informing selection in the following health technology contexts: diagnostic imaging evaluation [144]; vaccines evaluation [145–147]; evaluation of off-patent pharmaceuticals in emerging markets [148]; evaluation of pharmaceuticals in a development stage [149]; pharmaceutical pricing and reimbursement [150]; orphan drug reimbursement [151]; disinvestment and resource allocation processes [152]; hospital investment [153]; hospital decision-making [154]; value definitions [155]; priority setting [156, 157]; criteria beyond cost-effectiveness analysis [158]; defining equity [159]; and physiotherapy implementation [160].

Most structuring criteria studies (12) conducted literature reviews to inform criteria selection, while some of them combined reviews with surveys, interviews or workshops. Six studies used specific tools to structure or rank the criteria, namely direct scoring [148], the AHP [154], a discrete choice experiment [156], a design comparing technologies in clinical scenarios [144], predefined scales [158] and ELECTRE III [with ELECTRE standing for *ELimination Et Choix Traduisant la REalité* (ELimination and Choice Expressing REality)] [150].

Other reviewed studies with a main focus other than structuring criteria also devoted substantial work to structuring criteria in the following contexts: assessment of medical innovation [139], drugs [129] and new medicines [30], for setting up local HTA evaluation processes [140], for evaluating disease management programs [134], and rare disease interventions across disease areas [142].

#### (e) Modelling approach studies

Chen [161] developed an approach to deal with imprecise judgments. Broekhuizen and colleagues [162, 163] and Wen et al. [164] researched how to model uncertainty in patient preferences and/or clinical outcomes using MCDA combined with Monte-Carlo simulation. Three studies made use of the stochastic multi-criteria acceptability analysis approach (SMAA) to explore what can be concluded when limited or no preference information exists and a data-driven approach is used: one explored Mixed Treatment Comparison for evidence synthesis within SMAA [165]; a second proposed a stochastic multi-criteria discriminatory method based on SMAA to describe the likelihood of one treatment performing better than alternatives [166]; and, a third, presented the net monetary benefit framework as a special case of SMAA [167].

#### (f) Literature review studies

There are three studies in this category; the first, reviewed approaches adopted in 40 MCDA studies, analysing the objective of the study and lessons learned [21]. The second assessed 22 MCDA studies to analyse costs and benefits at different stages of medical innovation, reviewing the type of policy applications, methodology, and criteria used and respective definitions [22]. And, the third reviewed ten MCDA studies which involved patients in model building [36].

### Methodological quality of model applications

**Fig. 3:**
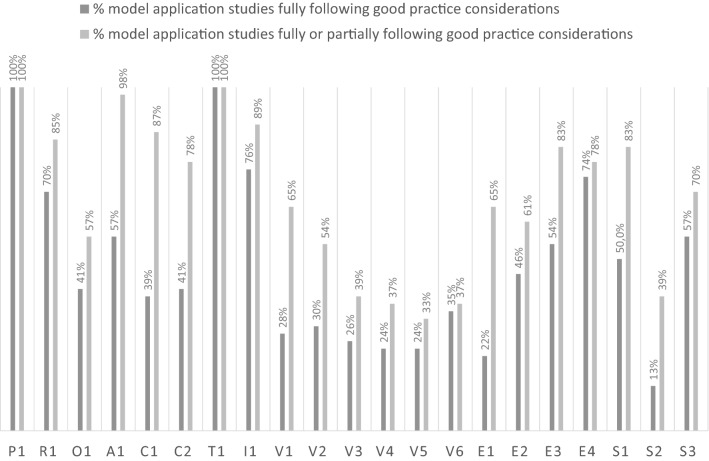
Percentage of studies fully or at least partly following good practice considerations in the PROACTIVE-S sub-steps. Note: Abbreviations in use are defined in the last column of Table 1. Source: the authors from the literature

The application of the PROACTIVE-S approach to assess the extent to which the 46 model application studies followed good methodological practice is shown in Table 2, where studies are classified as fully, partially or not complying with the PROACTIVE-S sub-steps (as defined in Table 1); Fig. 3 summarises results from the analyses.

**Table 2:**
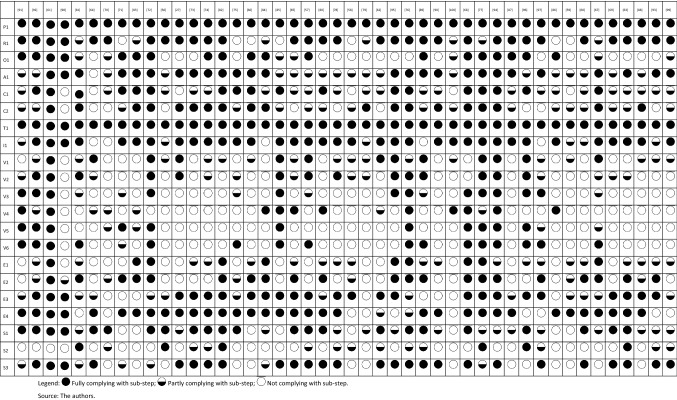
Adherence of 46 model application studies to the PROACTIVE-S framework and “the extent to which the study follows good practice considerations”

There are three broad areas of interest based on the data reported in Table 2 and the summary data in Fig. 3. First, the lowest levels of adherence to PROACTIVE-S’s good methodological practice considerations (≤ 50% of studies are fully or partly complying with good practice considerations) are found in the value measurement sub-steps and concern behavioural issues, such as: fully using methods for model building comply with multi-attribute decision theory (V3) (26% of studies); defining mechanisms to detect and correct inconsistencies (V4) (25% of studies); using procedures to model value functions (V5) (24% of studies); using weighting procedures that utilize weighting references and explain their rationale (V6) (35% of studies); and taking into consideration behavioural aspects (S2) (13% of studies).

Second, low levels of adherence (fully compliant at ≤ 50% and fully or partly compliant > 50%) are found in the following sub-steps: (fully) focusing on the objectives to be achieved (O1) (41% of studies); assessing relevant consequences in adequate attributes that comply with required properties and organising consequences of options into a performance matrix (C1) (39% of studies); discussing data sources and issues and consequences uncertainty (C2) (41% of studies); discussing model respect for exhaustiveness, non-redundancy and non-overlap properties in additive models or relevant properties for other models (V1) (28% of studies); discussing preference independence conditions and presenting the underlying model structure (V2) (30% of studies); explicitly modelling assumptions and the relevant uncertainties for the evaluation context (E1) (22% of studies); testing the consequences of model assumptions and uncertainty (E2) (46% of studies); and enabling replicability by making explicit model building participants, participatory scope and format, participatory timeline, and protocols of questioning (S1) (50% of studies).

Third, intermediate levels of adherence—translating into studies fully compliant with good methodological practice at > 50% but at a large distance from 100%—were found for the following sub-steps: studies fully defining and discussing the relevant health technologies to be evaluated and linked decisions (A1) (57% of studies); discussing model validation and requisiteness and questioning model results (E3) (54% of studies); and promoting participants’ reflection and iterativeness in model development while also promoting consensus and/or reflecting on ways to combine participants’ assessments (S3) (57% of studies).

Finally, higher but not full levels of adherence to good methodological practice were found in the following areas: studies fully discussing the perspectives of relevant stakeholders and key players, clarifying the perspective of the problem owner and discussing whose views should be considered in model building (R1) (70% of studies); distinguishing between evidence, options’ performance and value information (I1) (76% of studies); using computer technology to display results and motivate discussion (E4) (74% of studies).

### Reported limitations and challenges

The most common limitations and challenges that have been reported in the 129 reviewed studies are presented on Table 3. These were clustered by relatedness, leading to 12 concerns that were claimed by at least 4 studies each. These concerns relate to: (a) evidence and data processing within model building and use (47 studies, 36%); (b) differences in value systems and the influence of participants’ composition and numbers on evaluation (46 studies, 36%); (c) participants’ difficulties in understanding model building tasks and results (33 studies, 26%); (d) model developers having to trade-off methodological complexity with time and cost resources for model development (21 studies, 16%); (e) the selection of criteria and the construction of attributes in model structuring (20 studies, 15.5%); (f) modelling of uncertainty (19 studies, 15%); (g) addressing model additivity issues (17 studies, 13%); (h) the selection of methods (17 studies, 13%); (i) promoting consensus and dealing with the aggregation of participants’ answers (12 studies, 9%); (j) attempting to create universal/general evaluation models (9 studies, 7%); (k) fostering MCDA training and expertise (7 studies, 5%); and (l) generating model scores which have a meaningful interpretation (4 studies, 3%).

**Table 3:** Identification of challenges and limitations in published MCDA studies (aggregated into clusters; the descending order reflects the number of studies raising a particular challenge or limitation). Source: The authors from the literature

Cluster #	Summary (number of studies)	Clustered reported limitations and challenges	Articles expressing limitations and challenges
#1	Evidence and Data related Difficulties (TECHNICAL) (47 studies)	Multiple difficulties exist regarding the use of evidence and data in evaluation processes: information from multiple studies may be complex, not fully comparable (e.g. data often derived from separate trials differing in populations, treatment durations and calculated in different units), hard to capture by checklists or scales, have questionable quality, and participants may be overwhelmed by data (for instance numerous aggregations of data from non-standardised and often non-computerized databases). There may be lack of evidence and lack of good quality data for criteria deemed as relevant by evaluators, and there are challenges to synthesize relevant information. Participants may have a sense of information loss along evaluation processes. There is a lack of consensus about quantities such as ‘quality of life’ or ‘economic value’ of a healthy individual, which translate into evaluation difficulties. It is difficult to acquire and interpret data across heterogeneous health technologies	[18, 19, 21, 24–28, 31, 42, 60, 63, 69, 74, 80, 82–84, 87–89, 91, 94, 97, 98, 102, 103, 112, 113, 126, 130–132, 135, 137, 140, 142, 143, 151–153, 155, 158–160, 164, 165]
#2	Value System Differences and Participant Selection issues (SOCIAL) (46 studies)	There are variations in experts and stakeholders’ views and in the value systems of countries/regions/health systems. Value systems can vary over time and in response to new evidence. In multiple contexts evaluations rely on the views of members of a small panel/committee, with resulting evaluations being influenced possibly by participants’ characteristics and not being representative. In contexts where representativeness is important, it is not clear whose views should be considered. There are limits to involve a large number of participants in a face-to-face setting. MCDA studies have been involving much smaller numbers of participants than large patient preference studies	[19, 21, 24, 26, 27, 31, 33, 36, 57, 59, 60, 62, 63, 65, 66, 72, 73, 75, 78, 80, 82, 84, 97–99, 101–103, 106, 109, 111, 113, 116, 121, 123, 126, 129, 131, 132, 139, 142, 144, 147, 148, 153, 162]
#3	Participant Difficulties in Evaluation Processes (SOCIAL) (33 studies)	Participants face difficulties in interpreting data or in understanding evaluation processes; they also face cognitive difficulties in providing judgments (for instance using swing weighting, comparing mild and serious events, understanding orders of magnitude, interpreting weighting coefficients). Evaluation judgments may be frame-dependent (e.g. being influenced by the method in use), with multiple heuristics and biases. Applying, for instance, judgments may be prone to strategic behaviour, to vested interests and may be critically influenced by some participants; languages and translations may influence evaluations; participants typically have distinct levels of understanding; and weighting is influenced by ranges, with participants shying away from extremes	[18, 19, 21, 24, 26, 28, 30, 31, 33, 36, 43, 57–60, 68, 72, 73, 75–78, 96, 98, 99, 104, 106, 116, 144, 149–151, 155]
#4	Balancing Methodological Complexity and Resources (TECHNICAL) (21 studies)	There is methodological complexity in using MCDA in HTA and a trade-off between methodological complexity and MCDA resources (including costs and time for model development). Standards and requirements for MCDA modelling may limit flexibility, adaptability, and timeliness. Many MCDA models are simplistic, with the choice of simple, intuitive, easy to use techniques, even if there is a compromise on rigor; often only partial information is requested from experts because of cost and time. Analysts are faced with the trade-off between ensuring an exhaustive set of criteria and the time and cognitive effort associated with using more criteria. Time is needed for evaluators to get acquainted with MCDA processes, and there may be participant fatigue. A significant amount of work is involved in reporting a MCDA model to evaluate health technologies	[19, 24, 27, 28, 30, 33, 57, 59, 60, 69, 74, 97, 99, 116, 119, 127, 129, 140, 149, 151, 162]
#5	Criteria Selection and Attribute building Difficulties (TECHNICAL) (20 studies)	The definition of evaluation criteria and attributes is a long, difficult and subjective process, further complicated because of variability of HTA terminology adopted in the hospital context. Some criteria, such as equity, are difficult to operationalize and the use of attributes is open to subjective interpretations. There is a lack of guidelines on the number of criteria and the structure of the evaluation model can become too extensive if all criteria need to be taken into account, as well as it can lead to cognitive burden and time-consuming procedures. Several attributes can be chosen for a criterion and there are difficulties in defining references for those attributes. Work is needed to advise how to estimate baseline effects. Not all aspects to be accounted for are quantifiable and it may not be possible to incorporate them into an MCDA model (e.g. context issues related to system capacity and appropriate use of an intervention)	[21, 26, 30, 59, 61, 63, 73, 78, 82, 89, 123, 129, 130, 132, 134, 140, 149, 151, 157, 165]
#6	Uncertainty Modelling Needs (TECHNICAL) (19 studies)	Health technology assessment entails uncertainty from multiple sources, related to scoring and weighting methods, criteria choice and with attributes in use (such as some based on point systems), as well as with evaluation judgments. Evaluators may not be able to give exact preference information on weights. Several modelling options, such as the choice of time horizon for costs and benefits, can influence evaluation	[18, 19, 21, 24, 25, 33, 68, 75, 80, 93, 98, 113, 121, 122, 126, 143, 163, 164, 166]
#7	Model Additivity Issues (TECHNICAL) (17 studies)	There are multiple issues related to the use of an additive model—for instance, it cannot be used to deal with thresholds (e.g. a threshold of incidence of adverse events above which the drug is considered unacceptable), as one cannot use trade-offs with it. There is potential for criteria to be neither exhaustive nor mutually exclusive, there may be overlaps and double counting (e.g. some degree of overlap is inherent in many of the endpoints), and one needs to deal with interdependencies. More complex methods are known, but their adoption may imply that the elicitation questions become too hard for evaluators	[19, 21, 30, 32, 43, 61, 64, 77, 82, 93, 116, 121, 123, 133, 145, 151, 162]
#8	Methods’ Selection Issues (TECHNICAL) (17 studies)	There is no consensus about the best framework and about the best weighting method, and methods are not standardised which raises validity issues. The selection of method may introduce bias, and inadequate weighting practices are recognised in the literature. There is generally no gold standard against which to compare results and results have not been replicated by independent third parties. There is a sense of arbitrariness in the implementation of an MCDA approach. Acknowledging the existence of different schools of thought, the appropriate weighting technique is essential, as are the circumstances under which a specific technique should be used	[18, 26, 33, 43, 58, 68, 69, 98, 121, 122, 126, 128, 134, 146, 148]
#9	Consensus Promotion and Aggregation of Participant Answers (SOCIAL) (12 studies)	Despite the importance of promoting consensus, consensus agreement varies across studies and needs to be both accommodated and reported. There are also issues on how to properly combine individual judgments and to assess consensus	[18, 24, 26, 30, 99, 113, 116, 121, 131, 132, 142, 153]
#10	Introduce Flexibility Features for Universal/General Evaluation Models (TECHNICAL) (9 studies)	Despite the ambition of building universal (or general) models to compare distinct technologies across diseases and therapeutic areas, there are conceptual and methodological difficulties in developing such models. Different models tend to be built for different contexts	[24, 36, 59, 101, 103, 106, 121, 144, 155]
#11	MCDA Training and Expertise Needs (TECHNICAL) (7 studies)	There is a lack of familiarity with MCDA techniques and, consequently, there is a need for training staff for MCDA implementation, as well as a need for participant training (e.g. patient training). Training requires time and resources	[36, 43, 56, 84, 97, 101, 111, 116, 123, 136, 137]
#12	Model Scores Meaningfulness Issues (TECHNICAL) (4 studies)	There are difficulties in interpreting model outputs and in understanding the meaning of these outputs, which need to be tested and validated. Scores are relative and produced in an interval scale, and thus limit the usefulness of a cost-value ratio; they also do not provide information about the absolute effectiveness, utilities, or absolute costs in monetary units	[21, 36, 75, 93]

## Discussion

The objective of this paper was to review existing literature of MCDA applications in HTA using aggregation approaches, to identify the scope of research published to date, to develop an understanding of the challenges and limitations reported in published research and to assess the methodological quality of studies whose main focus was to develop multidimensional models to evaluate health technologies. Several messages can be taken from this review, including an understanding of how the application of MCDA in HTA literature needs to evolve to address the limitations and challenges identified in the 129 studies.

### Key messages

The systematic analysis of MCDA studies in the context of HTA has yielded seven key messages related to trends, the direction of published research to date and a range of methodological issues that need to be addressed in future research. These messages are discussed below.

Regarding trends in the application of MCDA in HTA studies, one observes a high growth in published research, with most studies developed in a small number of countries (UK, Netherlands, Canada, US, Germany and Italy) and disseminated through health (mainly non-clinical) journals. Model application studies are far from being able to cover all types of health technologies, decisions and decision contexts: evaluation models have been built mostly for pharmaceuticals and for general health technologies and interventions (although general technologies or interventions are restricted to one area, notably public health); most studies aimed to inform the selection of the best technology (with some looking into priority setting and resource allocation).

The majority of studies have developed experimental models in applied settings; the focus has been to discuss adoption, development and implementation issues and to develop frameworks for a wide range of contexts, thus showing the emerging and exploratory nature of research in the area. Studies discussing MCDA aspects have raised methodological issues and alerts for the methodological robustness of MCDA in the context of HTA, but also underlined the high potential and positive experience regarding MCDA exploration and adoption. Framework studies outlined MCDA processes for multiple technologies and evaluation purposes, and set principles, methods and processes for MCDA that address specific aspects and contexts.

The majority of studies made use of participatory approaches that relied on workshop sessions but other non-face-to-face and web-based processes have been explored, demonstrating the interest in overcoming the limitations of making decisions relying on a small number of individuals that meet face-to-face and the cost and time constraints.

Concerning methods in use, in light of the multicriteria value measurement literature, the fact that most studies did not model value functions and that AHP and point scaling were the two most commonly selected weighting procedures raises methodological issues (discussed below in the relevant section). Although issues concerning methodological quality have been raised in other HTA areas [168], our study findings provide evidence that more research, methodological quality improvements and more models developed in realistic settings are required. This is compatible with what others have argued in the literature [19, 32, 43, 121].

Regarding the analysis of the modelling application studies’ methodological quality in light of the PROACTIVE-S approach, results suggest action is needed to improve the methodological quality of MCDA in HTA studies. More than 50% of the studies had a number of shortcomings: they did not follow good practice regarding value measurement sub-steps, did not focus on objectives (not adopting a value-focused thinking perspective [34]), did not adopt best practice tools in building attributes to analyse health technology impact, did not address uncertainty in technology impact, did not model assumptions explicitly, did not detail social processes, and did not reflect upon behavioural issues. In general, the lowest methodological quality was found in value measurement sub-steps, as most studies expressed few concerns with model properties, dealt with preference independence and the underlying evaluation model structure, used methods that do not respect the theoretical foundations of multi-attribute value theory (mostly due to the use of the AHP technique that is prone to rank reversal [169, 170] and can violate a condition of order preservation [171] and due to the use of point systems that do not consider weighting references across criteria), did not address judgmental inconsistencies in model building, did not use proper procedures to measure partial value, and did not use adequate weighting procedures.

While authors may have conducted some methodological analyses but not expressed them in writing, overall results suggest that many model applications may not have been properly built and validated and, as a result, models may not have led to appropriate recommendations for decision-making. Detailed guidelines should be developed so that sound procedures and tools are adopted in model building. To ensure adequate methodological quality, published studies should include detailed methodological information so that they can be thoroughly analysed—for instance, studies should detail social processes in use for accuracy and for replicability, which may not be compatible with article size limitations defined in health journals. Some evidence points towards methodological issues not being identified during the article reviewing process: for instance, all studies presenting the formulation of an additive model should have made explicit the weighting references in use. These references are relevant to understand model results in light of the use of interval scales, and weighting coefficients can only be interpreted together with the references in use, as changes in references require weights to be recalculated. One study has described repeatedly the use of a ‘linear addictive equation’ [127]. Several studies have made use of similar modelling approaches, advocating the use of methods that do not necessarily follow good practice guidance.

Taking stock of the challenges and limitations identified in the reviewed studies, several challenges are identified by a very large number of studies. More than a quarter of the studies (a) raised questions regarding the use of health technology evidence and data in the evaluation process, (b) made explicit concerns regarding differences and variations in value systems across health stakeholders and contexts (expressing concerns about models reflecting the views of a small number of individuals and being influenced by the choice of individuals, such as committee members), and (c) discussed participants’ difficulties in model development and/or use. Given the scale of such concerns, it is imperative these aspects are addressed in future research. A discussion of how the “MCDA for HTA” literature can be developed to address each cluster of challenges and limitations follows, while the key issues are reported in Table 3.

### Addressing the challenges in and advancing the “MCDA for HTA” debate

#### Challenge 1: evidence and data-related difficulties

Forty-seven studies reported difficulties regarding the use of evidence and data in evaluation processes, particularly in synthesising information, in dealing with data non-comparability issues and with large volumes of data (complete description of cluster 1 in Table 3). While these issues also apply to HTA in general, for the “MCDA in HTA” context research can help to provide guidelines to address these issues; synthesis formats can be developed to capture what is regarded as key data (in line with evaluation objectives to be achieved with health technologies), by explaining how to address variability in technology impact assessment (e.g. through considering impact intervals and performing sensitivity analysis), and by designing model features that consider cases of data incomparability and lack of data among others. Regarding this last point, flexible and non-closed models can be explored in which attributes consider not only quantitative aspects but also qualitative assessments from evaluators (for instance, committee members). Such models have been explored in other contexts, e.g. in the case of faculty evaluation [172]: in this context it was deemed a critical feature for model adoption that evaluators should not only consider quantitative metrics but also other complementary aspects within a qualitative and non-closed but formal assessment (e.g. to consider the number of high quality publications and the number of citations from faculty members, evaluators could consider qualitative aspects such as prizes and adjust evaluation scores). This study context led to the combination of quantitative with qualitative assessments in a multiplicative model structure. Furthermore, there is scope for developing guidelines that clarify and characterise different types of uncertainties in data, and that explain clearly which procedures to undertake for each type of uncertainty, for instance departing from the work by Stewart and Durbach [173].

#### Challenge 2: value systems’ differences and participants’ selection issues

Forty-six studies were concerned with the variation of value systems across experts, stakeholders and health systems and over time, as well as with MCDA models in HTA relying on the views of a small number of participants that may not represent all relevant views. While it needs to be acknowledged that value systems change over time (being also influenced by new evidence), which implies that evaluation models need to be reassessed and updated occasionally, a wide range of concepts and tools can be developed to ensure that models have the potential to reflect the perspectives of a diverse and larger number of health stakeholders. To address this challenge, “socio-technical processes” can be effectively designed and tested to involve a larger and more representative number of HTA stakeholders in the evaluation of health technologies; this, for instance, would avoid having to rely solely on the perspectives of a small number of evaluation committee members. Such a path has already started to be explored in other health contexts. For example, within the scope of building a population health index (based on a multi-criteria model structure) to evaluate population health across European regions, a socio-technical approach was adopted combining non-face-to-face web-Delphi processes to collect the views of a large number of European experts and stakeholders with face-to-face decision conferencing processes with a strategic group for building the multi-criteria model (as informed by evidence and by the views collected in the web-Delphis) [174, 175]. These processes can be developed and adapted further to collect health stakeholder views to inform the building of MCDA in HTA models, having to consider consensus and other issues (explored further under challenge 9). Stakeholder theory and engagement literature (discussed in [176]) can help to clarify which stakeholders to involve, under which type of involvement (which may include informing, consulting and co-deciding involvements) and with which format. Additionally, social research studies have explored statistical concepts to inform which participant numbers by stakeholder group are required for representativeness [177].

If properly designed, developed and enhanced by technology, web-based processes can facilitate the collection of information from a larger number of participants at relatively low cost [178]; there may also be space to develop structured techniques to involve larger groups of people in face-to-face settings [178]. Approaches can be further developed so as to test whether value systems tend to change over time, following the research idea explored by Lienert et al. [179] through the study on the stability of preferences over time in the context of wastewater infrastructure decision-making. A few studies in the review have re-tested the preferences of those participating in MCDA modelling with such aim (e.g. [56]).

#### Challenge 3: participant difficulties in evaluation processes

Thirty-three studies have mentioned several types of participant difficulties in interpreting data, understanding evaluation processes, and providing judgments; additionally, they raised related behavioural issues and biases affecting the development of MCDA models in the context of HTA. MCDA can assist in providing friendly protocols to be tested in empirical applications. Studies can incorporate behavioural research features so as to test preferred modes of questioning (taking into account behavioural issues reported in MCDA development). Some methods have shown to be cognitively friendly in empirical settings [39]. For instance, several studies in health have been using the MACBETH approach [180–182] that provides an interactive questioning procedure based on qualitative judgements that only asks a decision-maker or a group for qualitative preference judgements between two elements at a time; this addresses cognitive uneasiness experienced by evaluators when trying to express their preference judgements numerically [54]; AHP also asked for qualitative judgments (on a ratio scale) in a large number of reviewed model applications, with studies providing positive feedback regarding its friendliness for participants. While several articles have reported participant difficulties in providing (quantitative) swing weighting judgments, MACBETH enables the use of qualitative swing weighting and has been used with a positive feedback in several of the reviewed model applications [61, 85, 94]. Other user-friendly and methodologically sound protocols may also be explored.

Behavioural research, informed by behavioural literature in general [53] and, specifically, by behavioural literature for MCDA contexts [55, 183], can be developed in MCDA for HTA, for example to compare participant preferences for modes of questioning, visual displays and methods. Eliminating bias from procedures that have been used in other contexts [184] can be adapted, as it is important for those facilitating the use of several protocols of questioning in the phase of model testing and validation: to illustrate, MACBETH qualitative swing weighting and quantitative swing weighting can be used interchangeably to explain and discuss the meaning of weighting coefficients to participants.

Training and guidelines for facilitation and making use of a wide range of existing resources [185–187] can be developed to assist those developing MCDA for HTA applications—facilitation skills can help managing participants and better communication in workshop settings. Other training issues are discussed in challenge 11, below.

#### Challenge 4: balancing methodological complexity and resources

Twenty-one studies reported concerns related to the methodological complexity of using MCDA in HTA and the need to balance methodological complexity with cost, time and cognition in model development. A first issue is that the HTA community is not fully acquainted with MCDA concepts, methods and tools, as in most cases HTA education and training programmes do not cover MCDA or cover it superficially. If MCDA is to progress convincingly in HTA, it is expected that these programmes will need to enhance their curricula by including MCDA topics, that more MCDA courses are offered, and that HTA experts wishing to apply MCDA collaborate closely with MCDA experts.

A second issue concerns the extent to which pragmatism is acceptable in model development, as simplification can lead to models that do not respect basic MCDA properties. Some of the reviewed studies accept model simplifications [19, 63, 74], explicitly opting to build simple attributes and use weighting protocols that do not comply with multi-attribute decision theory. Many model simplifications may be inappropriate and are invalid (this has been shown for instance in the case of for river rehabilitation [188]); to that end literature and guidelines should provide guidance on which simplifications are acceptable.

A third issue is that methodological complexity can be addressed with a higher focus on the design of the socio-technical process [45, 46] in line with: balancing evidence and participatory processes; balancing larger non-face-to-face interaction to collect the views of a larger number of individuals with smaller face-to-face participatory processes; and with preparing a wide range of materials to assist participants in helping to build technology evaluation models [189].

Finally, concerning the cost and time costs for model development, there is scope for developing frameworks to produce reusable or easily adaptable models for several decision *problematiques* [40] and generating templates, so that evaluators can follow good practice and balance time and effort. Different HTA *problematiques* require distinct types of modelling approaches to be made available to model developers, e.g. modelling for choosing the best health technology (such as the choice of best pharmaceutical [61]), modelling for ranking health technologies (such as the ambition to ordering intervention strategies [95]), modelling for classifying technologies (as in the case of deciding which pharmaceutical falls within a reimbursement category [57]), modelling for allocating resources (such as the need to allocate a commissioning budget or nursing time to health programmes [92, 190]), and modelling for optimizing health care processes, which are also classified as health technologies [5] (as in the case of simultaneously defining hospital and long-term service locations, size of facilities and referral networks [191–193] in line with the health system objectives).

#### Challenge 5: criteria selection and attribute construction difficulties

Twenty studies mentioned multiple issues and lack of guidance and support to the definition of evaluation criteria and to the construction of attributes. Guidelines to specifically assist in model structuring need to be developed, so as to avoid issues that should not arise if MCDA is properly used. Indeed, structuring is a key step in MCDA model development and results from applying the PROACTIVE-S approach suggest that researchers have not always adopted best practices or dedicated full attention to model structuring when developing model applications. If all relevant criteria are not considered and if attributes are inadequately designed, the succeeding steps in model development may not be successful [34] and claims of subjective interpretations of attributes may appear. A wide range of tools from the problem structuring methods literature can assist problem structuring for MCDA and generally have been unexplored in “MCDA for HTA” [194]. Clear examples on how to build and/or to model attributes may be developed, following [47, 195], with special attention to qualitative and multi-dimensional attributes that may be critical for cases with lack of data, of data incomparability and of preference dependence between criteria. New model structures should be researched so as to incorporate qualitative aspects within evaluations, and to explicitly deal with a lack or bad quality data (discussed in challenge 1). Existing literature explaining the pros and cons of choosing distinct reference levels within attributes—either local or global, absolute or relative levels [40, 48]—should be clearly made available to the “MCDA in HTA” research community. If a model becomes too large, hierarchical modelling techniques are also advised [196].

#### Challenge 6: uncertainty modelling needs

Nineteen studies raised issues related to model choice, methods in use, technology impact imprecision or variability, and participant judgments, which translate into different types of uncertainty. Although multiple studies have developed methods to deal with uncertainty and have clearly described different types of uncertainty [19], within the decision analysis spirit of ‘divide and conquer’ [197], there is scope for developing clear procedures on how to deal with each type of uncertainty so that participants and evaluators are better equipped with what modelling pathways to follow under the presence of each uncertainty source.

#### Challenge 7: model additivity issues

Seventeen studies raised issues related to the appropriateness of using an additive model, namely the way of dealing with thresholds (related to compensation of performance in the evaluation criteria), exhaustiveness of evaluation criteria, double counting (for instance related to the use of several endpoints), preference independence, and the potential cognitive burden related to the use of more complex methods. Clearer guidelines suggesting tests, protocols and tools may need to be developed in this area. Several modelling options may be explored to deal with non-compensability in the performance of technologies in distinct evaluation criteria, for instance, additive evaluation models can be combined with system rules in which minimal thresholds need to be attained so that the technology is considered for evaluation (use of thresholds such as in Bana e Costa et al. [196]). Concerning preference dependence, there is a need to develop tests with friendly protocols of questioning not only for identifying such issues (as in Oliveira et al.), but also to suggest how to make use of distinct model structures in a user-friendly way. Literature already advises on how to restructure models so as to respect additivity, for instance building constructed attributes that integrate preference dependent dimensions [48]; and some studies in health settings have already developed (and applied) user-friendly protocols of questioning to identify preference dependence cases and show how multilinear [195] and Choquet Integral-based models [198] can be built. Some studies in real settings have also explained the rationale for using multiplicative models [172]. Several of these studies have used the qualitative MACBETH protocol of questioning that has been shown to provide a user-friendly protocol of questioning [54, 181]. The structuring methods recalled in challenge 5 provide tools to avoid double counting and ensure exhaustiveness.

#### Challenge 8: method selection issues

Seventeen studies made explicit the desire to have a ‘best method and a best framework’ and raised validity and replicability issues. While it is not expected that there will be a single prevailing weighting method or approach in MCDA, there should be clarity about methods that have sound theoretical foundations and the limits of a pragmatic MCDA (discussed in challenge 4). Those developing MCDA for HTA should consider using several protocols during model development and validation (discussed in challenge 3), so as to ensure that evaluations do not rely on methods and that participants develop a better understanding about the evaluation model and results. Behavioural research (discussed in challenge 2), may be carried out to test whether participants prefer to express judgments in specific formats and under distinct methods, and to gauge the best forms to communicate model outputs. Further procedures for model testing and validation can be developed, for example involving experimental design in comparing model evaluations with real decisions within an ex-post evaluation frame. Replication of studies, analysis of preference stability and model retests (addressed in challenge 2) can also be used.

#### Challenge 9: consensus promotion and aggregation of participants’ answers issues

Twelve studies have explicitly recognised the importance of promoting consensus. It has been observed that consensus levels vary across studies and that clarity is needed about how to combine individual judgments. Following the view that a health technology evaluation model should be requisite (based on Phillips [49], it should be ‘sufficient in form and content to resolve the issues at hand’), the socio-technical design of the model building process needs to incorporate concepts and tools from group decision-making—for instance on voting systems, group decision support systems, group facilitation, and group thinking modelling (multiple issues covered in Kilgour and Eden [199])—to promote collaboration, convergence and alignment in model building [45]. The combination of individual judgments is relevant either in face-to-face contexts in which participants express their preferences that need to be combined and visualised by the group, and in non-face-to-face contexts, which require aggregation of individual answers. The choice of either format or their combination within a collaborative value modelling framework (such as proposed in [200]) may depend on time, cost and participant availability. To that end, there is a need for tools and guidance on how to proceed in such contexts and how to summarise and aggregate individual judgments or scores. Again, behavioural research can help answer which settings are more effective to promote consensus.

#### Challenge 10: introduce flexibility features for universal/general evaluation models

Nine studies have raised questions about whether it is possible to build general models that can be used to compare distinct health technologies across diseases or therapeutic areas. Despite the multitude of issues related to the evaluation of distinct technologies—for instance in comparing endpoints across diseases—it is open to “MCDA in HTA” research to introduce flexibility features in evaluation models and, thereby, promote their use across contexts. Such features include exploring: (a) the use of equivalence attributes (following the concept of strategic equivalence as defined in Keeney and Raiffa [16]), so that an attribute can be defined differently for different diseases but that it can be simultaneously compared across diseases; (b) the use of absolute references within each attribute that can be translated for distinct contexts (as discussed in challenge 5); (c) the use of qualitative assessments to complement quantitative assessments (as discussed in challenges 1 and 5); and (d) the use of weighting intervals that enable adjustment of weights for the context. The studies analysed within the review have most commonly used simple additive models, but some of these suggestions have been explored in other contexts, such as in Bana e Costa and Oliveira [172] in the context of faculty evaluation (notably the following figures were explored: qualitative assessments by evaluators; and interval weighting combined with optimization so that each faculty member has the combination of weighting coefficients that maximizes their value score).

#### Challenge 11: MCDA training and expertise needs

Seven studies have raised explicit concerns regarding the familiarity with MCDA techniques and the need for training researchers and participants. As discussed above, training and education in HTA rarely considers MCDA topics and user-friendly materials—such as videos—explaining the scope, features and applicability of MCDA in HTA are scarce. Successful and unsuccessful cases of application and of real implementation should be communicated clearly. Additionally, MCDA in the context of HTA should develop specifically designed decision support tools [201] to enable proper development of health technology evaluation models. Research can also explore the connection between MCDA and other evaluation techniques (for instance, Postmus et al. [202] have explored the extent to which net monetary benefit is a special case of SMAA).

#### Challenge 12: model scores and meaningfulness issues

Four studies have discussed issues related to the interpretation of model outputs and the meaning of model scores. These aspects relate to the use of interval scales, as multicriteria models based upon simple additive models produce value scores for health technologies that need to be anchored in two reference levels—for instance 100 and 0 corresponding to the best or worst plausible performances, respectively. These references are critical not only for weighting but also for the interpretation of value scores (for instance, what does a zero value mean?). This choice of reference levels relates also to issues discussed in challenge 5 (e.g. use of global or local attribute scales), and several paths may be explored to enable a meaningful interpretation of value scores. First, there are modelling features that can be used in some contexts—such as the use of absolute, intrinsic and meaningful references within attributes—that promote an understanding and interpretation of model scores. Second, when two technologies are compared, following the logic of the incremental cost effectiveness ratio (ICER), it is always possible to take zero (or placebo in economic evaluation) as the comparator. Based on this, analyses can be performed regarding the added cost and the added value on a common scale.

**Fig. 4:**
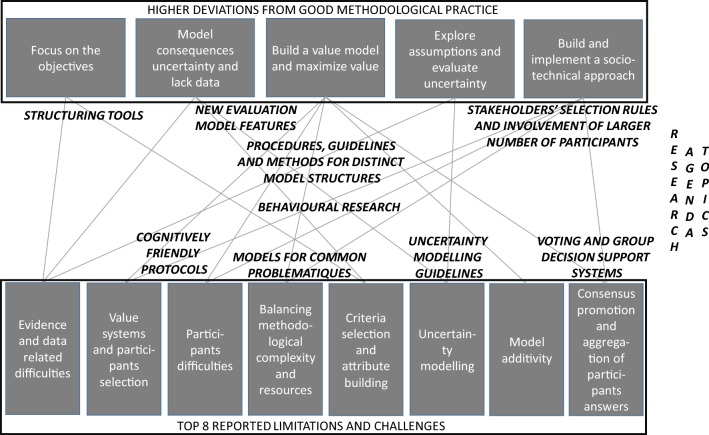
Interconnectedness between the MCDA modelling steps with higher deviations from good methodological practice (on top), and the eight most reported limitations and challenges reported in MCDA in HTA studies (on bottom). Lines in the middle depict interrelations between those deviations and limitations/challenges, with topics near the lines (in capital letters) synthesising areas relevant for developing the state of the art within MCDA for HTA (topics discussed along the “Discussion” section). Source: the authors from the literature

Figure 4 displays a visual representation linking the areas in which model application studies most highly deviating from methodological good practice with the 8 most important methodological challenges expressed in studies; it also displays suggested topics for research, which may address improvements in methodological practice or in reported limitations and challenges more explicitly and directly. There can be a connection between methodological challenges and deviations from methodological quality perspective and that suggested research topics have the potential to simultaneously contribute to good methodological robustness and to help researchers working in the area.

### Study limitations

The study is not without limitations and challenges. First, given the multiple designations and the variety of nomenclature adopted in MCDA studies relevant to HTA, the choice of terms may have affected some of the results and potentially relevant studies may not have been included; additionally, restricting analysis to journal articles and book chapters published in English may also have had some impact on results. However, our search strategy has been comprehensive enough to ensure that the likelihood of omitting an eligible study was small. Despite that, and considering the recent upward trend in the publication of relevant studies over the past 5 years, it is likely that in the very near future an increasing number of new studies will be published, but we cannot control for this.

Second, there seems to be a different understanding on what MCDA actually is among scientists developing studies in this particular field. For instance, there are studies included in the sample collecting information from participants through surveys—without further interacting with participants or testing and validating models—and describing that they have been developing MCDA evaluation models. A strict view on what is MCDA could mean that these studies should be excluded. However, as these studies are insightful in many other respects, such as on how to involve participants in the evaluation of health technologies and on which areas it is relevant to explore MCDA in HTA, we decided to include them. Furthermore, this decision is also coherent with the objective of analysing the methodological quality of studies in the area.

## Conclusion

This study shows that the application of MCDA in HTA is a growing field with increasing numbers of studies exploring its use in multiple contexts and under distinct perspectives, embedding its concepts and methods within technology policy- and decision-making processes, and showcasing its usefulness. Results show a number of limitations and challenges to address, a need to develop research and guidelines to promote quality and scientific rigor in the use of MCDA in HTA, as well as scope for advancing robust methodologies, processes and tools to assist modellers in the use of methods.

Several research paths have been identified within the scope of this study as potentially addressing the identified methodological challenges. Such paths include developing specific modelling approaches to account for distinct decision HTA contexts, such as to inform adoption, reimbursement and pricing decisions. In a way similar to HTA, training and education tools need to be developed and made available. To address concerns made explicit by researchers regarding the use of evidence and data within multicriteria modelling, new studies need to explore standardised ways of synthesising quantitative evidence and data as well as capture the quality of evidence in a structured format. Such synthesis formats should also be aligned with the objectives to be attained in the evaluation context. Additionally, studies in the area need to balance social with technical aspects in model development, and those interested in applying MCDA in the HTA context should learn from best practice and the experience from those developing models in practical settings. Collaborative research involving multiple health stakeholders is needed, and new technologies with a potential to involve and collect the views from a larger number of perspectives at a lower cost may be carefully designed and tested.

## Appendix A

**Table:** Adopted search protocol for the systematic review of MCDA studies in HTA, with the following rules being adopted: [(“A” or “B” or “C” or “D”) and (“E” or “F” or “G” or “H”]

A: “MCDA” OR “Multicriteria Decision Analysis” OR “Multi-criteria Decision Analysis” OR “Multi Criteria Decision Analysis” OR “Multiple Criteria Decision Analysis” OR “Multiple-Criteria Decision Analysis” B: “Multicriteria Analysis” OR “Multi-criteria Analysis” OR “Multiple-criteria” C: “MAVT” OR “MAUT” OR “Multiattribute Decision Theory” OR “Multi-attribute Decision Theory” OR “Multiattribute Utility Theory” OR “Multi-attribute Utility Theory” OR “Multiattribute Utility” OR “Multi-attribute Utility” D: “Multicriteria Decision Aiding” OR “Multiple-criteria Decision-Making” OR “Multiple criteria Decision-Making” OR “Multicriteria Decision-making” OR “Multiple-characteristics decision-making” OR “MCDM” E: “Multicriteria Resource Allocation” OR “Multiple Criteria Resource Allocation” OR “Portfolio Decision Analysis” OR ((“Multicriteria Optimization” OR “Multiple Criteria Optimization”) AND “Resource Allocation”)	F: “HTA” OR “Health Technology Assessment” OR “Health Technology Appraisal” OR (“Health” AND “Technology” AND “Evaluation”) OR (“Health” AND “Technologies” AND “Evaluation”) OR (“Benefit-risk Assessment” AND “Health”) OR (“Value-based Assessment” AND “Health”) OR (“Economic evaluation” AND “Health”) G: (((“Medical” OR “Clinical” OR “Hospital” OR “Health”) AND (“Devices” OR “Equipment” OR “Technology”)) OR “Drugs” OR “Pharmaceutical” OR “Medicine” OR “Screening” OR “Surgical”) AND (“Benefit-risk” OR “Appraisal” OR “Valuation” OR “Assessment” OR “Value Measurement” OR “Value-based Assessment”) H: (“Treatment” OR “Therapy” OR Interventions” OR “Intervention”) AND (“Health” OR “Clinical” OR “Medical”) AND (“Benefit-risk” OR “Appraisal” OR “Valuation” OR “Assessment” OR “Value Measurement” OR “Value-based Assessment”)
